# Cross-Species Functional Conservation and Possible Origin of the N-Terminal Specificity Domain of Mitochondrial Presequences

**DOI:** 10.3389/fpls.2020.00064

**Published:** 2020-02-13

**Authors:** Dong Wook Lee, Sumin Lee, Chan-Ki Min, Cana Park, Jeong-Mok Kim, Cheol-Sang Hwang, Sang Ki Park, Nam-Hyuk Cho, Inhwan Hwang

**Affiliations:** ^1^Division of Integrative Biosciences and Biotechnology, Pohang University of Science and Technology, Pohang, South Korea; ^2^Department of Integrative Food, Bioscience and Biotechnology, Chonnam National University, Gwangju, South Korea; ^3^Department of Bioenergy Science and Technology, Chonnam National University, Gwangju, South Korea; ^4^Department of Life Sciences, Pohang University of Science and Technology, Pohang, South Korea; ^5^Department of Microbiology and Immunology, Seoul National University College of Medicine, Seoul, South Korea

**Keywords:** chloroplast, mitochondria, transit peptide, presequence, N-terminal specificity domain, TAT (twin-arginine translocation) signal sequence, evolution of endosymbiotic organelles

## Abstract

Plants have two endosymbiotic organelles, chloroplast and mitochondrion. Although they have their own genomes, proteome assembly in these organelles depends on the import of proteins encoded by the nuclear genome. Previously, we elucidated the general design principles of chloroplast and mitochondrial targeting signals, transit peptide, and presequence, respectively, which are highly diverse in primary structure. Both targeting signals are composed of N-terminal specificity domain and C-terminal translocation domain. Especially, the N-terminal specificity domain of mitochondrial presequences contains multiple arginine residues and hydrophobic sequence motif. In this study we investigated whether the design principles of plant mitochondrial presequences can be applied to those in other eukaryotic species. We provide evidence that both presequences and import mechanisms are remarkably conserved throughout the species. In addition, we present evidence that the N-terminal specificity domain of presequence might have evolved from the bacterial TAT (twin-arginine translocation) signal sequence.

## Introduction

The mitochondrion and chloroplast are thought to have evolved from α-proteobacteria and cyanobacteria, respectively, by endosymbiosis (Gray, 2012). A prerequisite for the conversion of endosymbiotic bacteria to organelles is the development of new mechanisms for protein targeting from the cytosol to the newly evolving organelles (Dyall et al., 2004; Gross and Bhattacharya, 2011).

Eukaryotic cells can be divided into two groups, one with both endosymbiotic organelles and the other with only mitochondria. Plant cells belong to the first group, with both organelles. Thus, plant cells should have distinct mechanisms for protein targeting to the chloroplast and mitochondria (Schleiff and Becker, 2011; Garg and Gould, 2016). Indeed, we recently found that chloroplast transit peptides and presequences consist of two functionally different domains in plant cells (Lee et al., 2019). The N-terminal specificity domain confers import specificity, whereas the C-terminal domain is interchangeable between the transit peptide and presequence, and functions in the translocation of organellar proteins across the envelope membranes (Lee et al., 2019). The N-terminal specificity domain of the mitochondrial presequence contains two sequence elements: multiple arginine residues and a hydrophobic sequence motif (Abe et al., 2000; Lee et al., 2019). Arginine residues play a crucial role in another signal sequence known as the twin-arginine translocation (TAT) signal sequence (Alami et al., 2002; Cline, 2015). Similar to the arginines in the TAT signal sequence, arginine residues in presequences cannot be functionally replaced with another positively charged residue, lysine (Lee et al., 2019). In the TAT signal sequence, another important sequence motif is the hydrophobic (h-) region downstream of the conserved twin-arginine (RR) motif (Ulfig et al., 2017). In fact, the presequence contains a hydrophobic region identified as a Tom20-binding consensus motif (Abe et al., 2000; Lee et al., 2019). Thus, the N-terminal specificity domain of the presequence shows a high degree of similarity to the TAT signal sequence with respect to its sequence motifs. However, it is still not understood how presequences of mitochondrial proteins evolved during the endosymbiotic conversion of α-proteobacteria to mitochondria. In this study, we examined 1) whether the mechanism by which plant presequences confer mitochondrial targeting specificity also applies to presequences of animal and fungal proteins, and 2) whether TAT signal sequences can be functional substitutes for the N-terminal specificity domain of mitochondrial presequences.

## Materials and Methods

### Plant Materials and Growth Conditions

*Arabidopsis thaliana* (Colombia ecotype) was cultivated on Gamborg B5 plates (G0210.0050; Duchefa, Haarlem, Netherlands) under 40% relative humidity, 22°C, and a 16-h light/8-h dark cycle in a growth chamber. Protoplasts were prepared from leaf tissues of 2- to 3-week-old plants.

### Plasmid DNA Construction and PCR-Based Mutagenesis

DNA fragments containing protein import signals were PCR-amplified from genomic DNA or cDNA of *Arabidopsis* and human cells using gene-specific primers. For transient expression analysis in *Arabidopsis* protoplasts, PCR products were digested with restriction endonucleases and subcloned into the pUC-GFP containing the cauliflower mosaic virus 35S promoter, GFP, and Nos terminator. For transfection and immunocytochemistry in HEK293 or HeLa cells, DNA fragments were subcloned into the pEGFP-N1 or N2 vector.

### PEG-Mediated Transformation and *In Vivo* Targeting of Reporter Proteins

Plasmid DNA used for PEG (polyethylene glycol)-mediated transformation was purified using Qiagen columns (Qiagen). DNA was introduced into *Arabidopsis* protoplasts by the PEG-mediated transformation method, as described previously (Jin et al., 2001; Lee and Hwang, 2011). Images of transformed protoplasts were acquired, as described previously (Jin et al., 2001; Lee and Hwang, 2011). To define the localization of transformed constructs, more than 50 GFP-positive cells in each transformation were observed. The localization pattern observed from more than 95% of GFP-positive protoplasts was regarded as the representative localization in this study.

### Transfections and Immunocytochemistry

HEK293 or HeLa cells were grown in Dulbecco's modified Eagle medium supplemented with 10% fetal bovine serum and antibiotics under 5% CO_2_ at 37°C. Cells were transfected using Lipofectamine 2000 according to the manufacturer's instructions. Plasmid DNAs were mixed with the reagents in Opti-MEM media (Invitrogen), and the mixture was layered over cells. At 12 h after transfection, the incubation media were replaced with fresh media and further incubated for 24 h. HEK293 cells were fixed in 4% paraformaldehyde in phosphate-buffered saline (PBS) for 15 min and incubated in blocking solution (2% goat serum and 1% Triton X-100 in PBS) for 20 min. Cells were incubated with rabbit anti-GFP (1:2,000, Molecular Probes) and mouse anti-Tom20 antibodies (1:100, Santa Cruz Biotechnology) for 1.5 h at room temperature, followed by incubation with Alexa Fluor 488-conjugated goat anti-rabbit IgG and Alexa Fluor 568-conjugated goat anti-mouse IgG secondary antibodies (Molecular Probes), respectively, for 1 h at room temperature. Cells were washed with PBS three times and mounted in antifade medium (Molecular Probes). Images were taken using an LSCM (FluoView-FV1000, Olympus). To define the localization of transformed constructs, more than 50 GFP-positive cells in each transformation were observed. The localization pattern observed from more than 95% of GFP-positive protoplasts was regarded as the representative localization in this study.

### Yeast Transformation

The constructs *RbcS[N49]:yeGFP* and *TorA[9−42]RbcS[43−79]:yeGFP* were generated in the yeast/*E. coli* shuttle vector pRS316 (CEN/ARS, URA3), which contains the constitutive TDH3 promoter. Constructed plasmids were transformed into yeast strain JD53 (MATα trp1Δ63 ura3-52 his3Δ200 leu2-3, 112 lys2-801) using the lithium acetate/single-stranded carrier DNA/polyethylene glycol (LiAc/SS-Carrier DNA/PEG) method (Gietz and Woods, 2006). Yeast transformants were selected and cultured in synthetic complete medium (0.17% yeast nitrogen base, 0.5% ammonium sulfate, 2% glucose, 0.06% drop-out mixture for a given auxotrophic strain) without uracil.

### Western Blotting

To prepare total protein extracts from transformed protoplasts or mammalian cells, transformed cells were lysed in the buffer (50 mM Tris-HCl, pH 7.5, 150 mM NaCl, 1 mM EDTA, 1% Triton X-100, 1× protease inhibitor cocktail) by sonication, followed by centrifugation at 3,000×*g* for 10 min. The supernatants were mixed with SDS loading buffer and boiled for 5 min. To prepare total protein extracts from transformed yeast cells, transformed cells were resuspended in the buffer (0.1 M NaOH, 50 mM EDTA, 2% SDS, 2% β-mercaptoethanol), followed by sonication. After centrifugation at 3,000×*g* for 10 min, the supernatants were mixed with SDS loading buffer and boiled for 5 min. For western blotting, protein samples were separated on 10% polyacrylamide gels for SDS-PAGE, followed by the transfer onto PVDF (polyvinylidene difluoride) membrane. PVDF membranes containing proteins were incubated with anti-GFP antibody (Clontech, 1:5000 dilution) at 4°C for 6 h. After washing for 10 min three times with TBS-T (Tris-buffered saline, 0.1% Tween 20) buffer, membranes were incubated with HRP-conjugated anti-mouse secondary antibody at 4°C for 4 h. After washing for 10 min three times, western blot images were captured using LAS-4000 (GE Healthcare Life Sciences).

## Results

### Conservation of the Mitochondrial Protein Import Mechanism in Animal and Plant Cells

**Figure 1:**
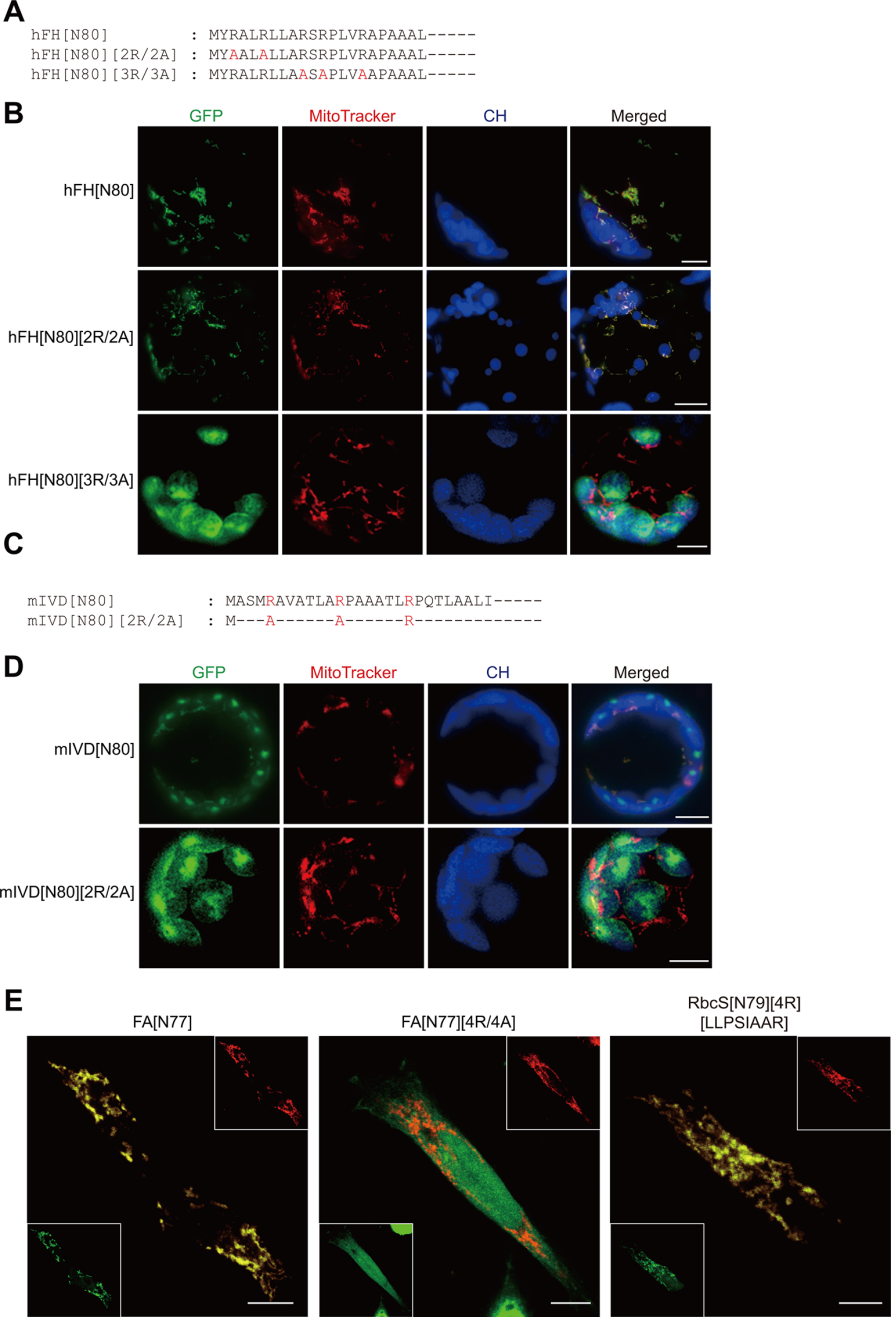
Presequence-mediated protein import into mitochondria is conserved in plant and non-plant cells. **(A**, **C)** Sequences of hFH[N80], mIVD[N80], and their alanine-substitution mutants. **(B**, **D)** Localization of reporter proteins. Green, red, and blue signals represent GFP, mitochondria stained with MitoTracker red, and chlorophyll autofluorescence, respectively. Scale bar = 20 μm. **(E)** Localization of reporter proteins in HEK293 cells. The indicated constructs were transiently expressed in HEK293. GFP and hTom20 were detected using anti-GFP and Tom20 antibodies, respectively. Scale bar = 10 µm.

We first examined whether presequences of animal and fungal mitochondrial proteins can support protein import into mitochondria in plant cells. Similar to plant presequences, the presequence of human mitochondrial fumarate hydratase (hFH) also contained five arginines in the N-terminal region (**Figure 1A**) (Dik et al., 2016). To test whether hFH[80] functions as a presequence in plant cells, it was fused to GFP. The resulting construct, *hFH[N80]:GFP*, was introduced into *Arabidopsis* protoplasts and the localization of GFP was examined by fluorescence microscopy. The staining pattern overlapped with the staining pattern of MitoTracker red (**Figure 1B**), indicating that hFH[80] delivers proteins into mitochondria in plant cells. In a previous study, we showed that the substitution of multiple arginines with alanines in the presequence leads to protein import into chloroplasts (Lee et al., 2019). Human mitochondrial fumarate hydratase (hFH) contains five arginine residues in hFH[N16] (**Figure 1A**). We replaced the first two or last three arginine residues of hFH[N80] with alanine residues to produce hFH[N80][2R/2A] or hFH[N80][3R/3A], respectively (**Figure 1A**). These mutant presequences were fused to GFP, and the resultant constructs were introduced into protoplasts. hFH[N80][2R/2A] supported protein import into mitochondria, as indicated by the colocalization of GFP with MitoTracker red in *Arabidopsis* protoplasts. By contrast, hFH[N80][3R/3A] delivered proteins into chloroplasts (**Figure 1B**), indicating that the last three arginines are crucial for mitochondrial specificity. Next, using the mitochondrial presequence of Isovaleryl-CoA dehydrogenase of *Magnaporthe oryzae* (mIVD), a blast fungus (Li et al., 2019), we found that mIVD[N80] delivered proteins into both chloroplasts and mitochondria in *Arabidopsis* protoplasts (**Figures 1C, D**), indicating that mIVD[N80] is less specific to mitochondria in plants compared with hFH[N80]. However, its R/A substitution mutants, mIVD[N80][2R/2A], showed exclusive chloroplast import (**Figures 1C, D**), indicating that the arginine residues of mIVD[N80] are critical for protein import into mitochondria in plants. Taken together, these results indicate that presequences of animal and fungal proteins behave more or less the same way in plants.

Next, using a reciprocal approach, we examined the behavior of the plant presequence FA[N77] which is a presequence of Arabidopsis F1-ATPase γ-subunit and its substitution mutants in animal cells (**Figure 1E**) (Lee et al., 2012; Lee et al., 2019). In animal cells, FA[N77] supported protein import into mitochondria, whereas FA[N77][4R/4A] lacking multiple arginines did not, confirming the importance of multiple arginine motifs for protein import into mitochondria in animal cells. In a previous study, we used an RbcS (Rubisco small subunit) transit peptide, RbcS[N79], to generate an artificial mitochondrial presequence, RbcS[N79][4R][LLPSIAAR], by introducing four arginine residues (4R) and a hydrophobic sequence motif (LLPSIAAR) from FA[N79]. The artificial sequence RbcS[N79][4R][LLPSIAAR] supports protein import into mitochondria in *Arabidopsis* protoplasts (Lee et al., 2019). The sequence also supported protein import into mitochondria in animal cells, despite the fact that other than the two motifs, it is a functional transit peptide (**Figure 1E**). These results indicate that the introduction of two mitochondrial specificity motifs converts a functional transit peptide to a presequence in animal cells, as in plant cells. Taken together, these results suggest that the mechanism underlying mitochondrial protein import is conserved in animal, fungal, and plant cells.

### Bacterial Twin-Arginine Translocation Sequences Functionally Replace the N-Terminal Specificity Domain of Presequences

**Figure 2:**
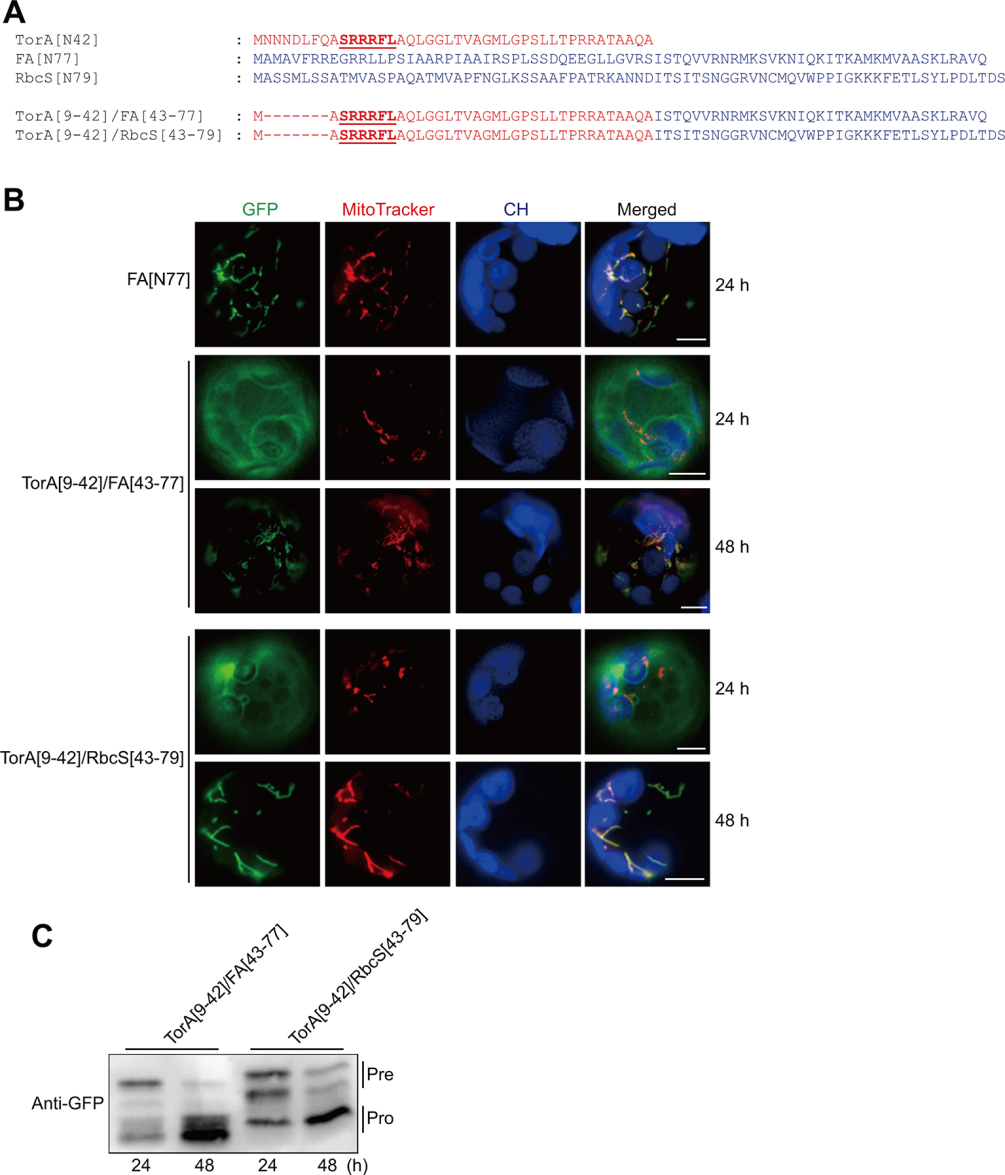
TorA[9–42] confers mitochondrial specificity to transit peptides or functionally replaces the N-terminal domain of presequences in plant cells. **(A)** Sequences of TorA[N42], RbcS[N79], FA[N77], and their hybrid constructs. **(B)** Localization of reporter proteins. Green, red, and blue signals represent GFP, mitochondria stained with MitoTracker red, and chlorophyll autofluorescence, respectively. Scale bar = 20 μm. **(C)** Western blot analysis of reporter proteins. Protoplast extracts were analyzed by western blotting using anti-GFP antibody.

The results described above further supported the importance of multiple arginine residues and the neighboring hydrophobic region in the presequence for protein import into mitochondria, not only in plants but also in other eukaryotic species. To determine the evolutionary origin of the presequence, we examined whether similar sequence motifs are used in other targeting signals. Highly similar motifs are found in TAT signal sequences in bacteria and chloroplasts. Similar to the mitochondrial specificity motifs, the twin-arginine residues are invariant and cannot be replaced with lysines (Alami et al., 2002; Cline, 2015). We asked whether the TAT signal sequence can functionally replace the N-terminal specificity domain of the presequence and change a transit peptide to a presequence. We chose the TorA (trimethylamine N-oxide reductase) TAT signal sequence (Frobel et al., 2011) for this purpose. Since TorA has a long N-terminal region in front of twin-arginine residues, we used TorA[9–42] to replace 42 amino acids of FA[N77] and RbcS[N79], yielding TorA[9–42]/FA[43–77] and TorA[9–42]/RbcS[43–79], respectively (**Figure 2A**). These constructs were transformed into *Arabidopsis* protoplasts. At 24 h after transformation, both constructs were mainly localized in the cytosol (**Figure 2B**). However, at 48 h after transformation, they were exclusively localized to mitochondria (**Figure 2B**), indicating that the hybrid sequences support protein import into mitochondria at a lower efficiency. To confirm these results biochemically, total protein extracts from transformed protoplasts were analyzed by western blotting using an anti-GFP antibody. At 24 h after transformation, we detected these constructs at the band positions corresponding to the precursor forms. At 48 h after transformation, we detected both proteins at the band positions corresponding to the processed form. These biochemical results are consistent with the GFP patterns in the protoplasts (**Figures 2B, C**). To corroborate this finding, we evaluated another TAT signal sequence of NapA (nitrate reductase) (Dow et al., 2014). The N-terminal 34 amino acids of the FA presequence were replaced with NapA[1–34] to generate NapA[1-34]/FA[35–77] (**Supplementary Figure S1A**). At 48 h after transformation, this hybrid sequence delivered protein into mitochondria (**Supplementary Figure S1B**). Next, RbcS[N79] and PORA[N80], the transit peptide of protochlorophyllide oxidoreductase A, were replaced with NapA[1–34] to generate NapA[1–34]/RbcS[35–79] and NapA[1–34]/PORA[35–80], respectively (**Supplementary Figure S1C**) (Lee et al., 2008; Lee et al., 2019). At 48 h after transformation, NapA[1–34]/PORA[35–80] was exclusively localized to mitochondria, whereas NapA[1–34]/RbcS[35–79] was dually targeted to both chloroplast and mitochondria (**Supplementary Figure S1D**).

**Figure 3:**
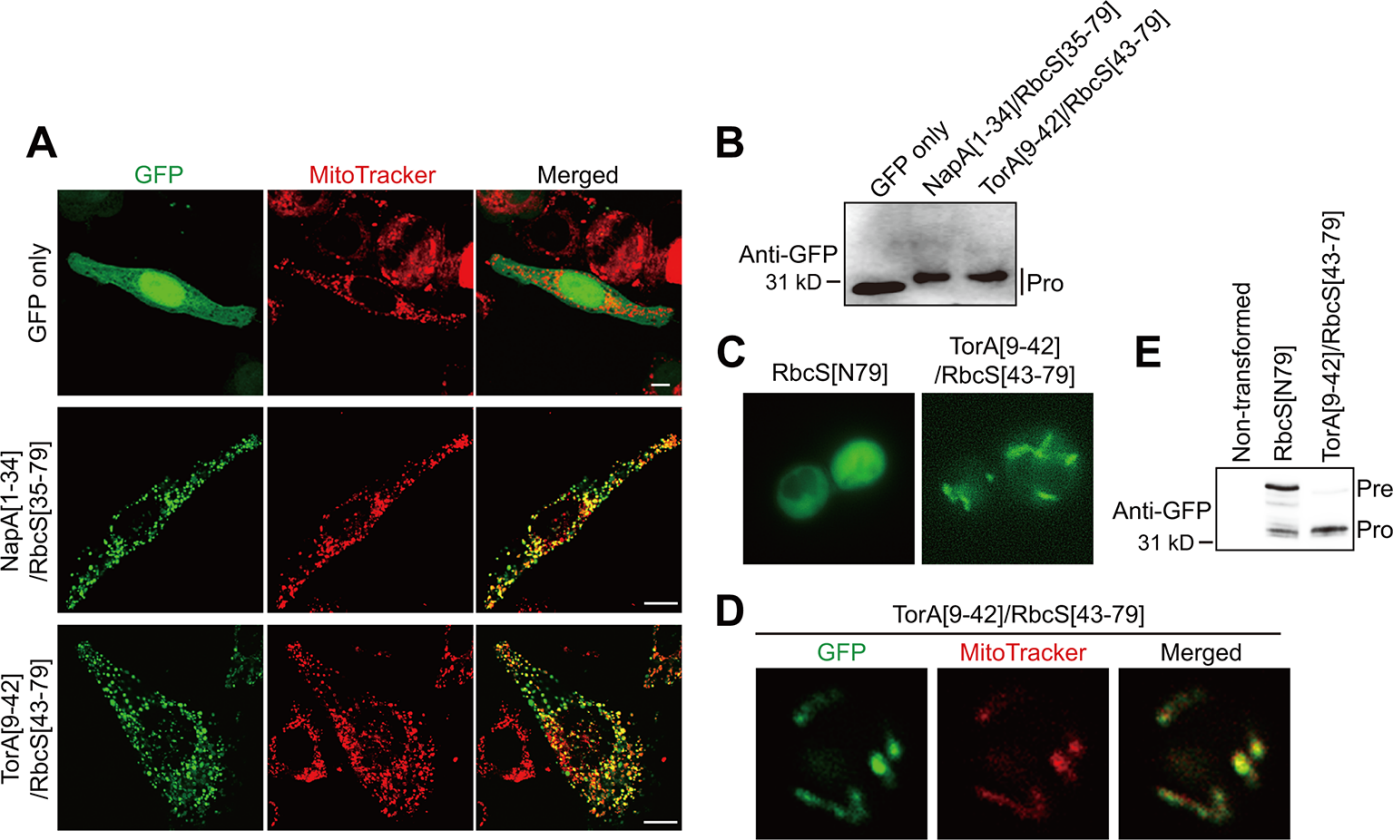
TAT sequences functionally replace the mitochondrial specificity domain in presequences and confer mitochondrial specificity to transit peptides in non-plant cells. **(A)** Localization of reporter proteins in HeLa cells. EGFP, control without presequence. Scale bars = 10 μm. **(B**, **E)** Western blot analysis of reporter proteins in HeLa cells and yeasts. Pre, precursor form; Pro, processed form. **(C**, **D)** Localization of reporter proteins. Green and red signals represent GFP and mitochondria stained with MitoTracker red, respectively.

We then tested whether TAT sequences confer mitochondrial specificity in other eukaryotic cells. HeLa cells were transformed with *GFP* only as a control, *NapA[1–34]/RbcS[35–79]*, or *TorA[9–42]/RbcS[43–79]*. At 12 h after transfection, both hybrid constructs supported efficient protein import into mitochondria (**Figures 3A, B**), indicating that the incorporation of TAT signal sequences into transit peptides produces functional presequences for animal cells. We further tested TAT/transit peptide hybrid sequences for mitochondrial import in a single-celled eukaryote, *Saccharomyces cerevisiae*. In yeast, TorA[9–42]RbcS[43–79], but not RbcS[79], supported mitochondrial import, confirming that the incorporation of the TAT signal sequence to chloroplast transit peptides produces functional presequences in yeast (**Figures 3C–3E**).

## Discussion

In this study, we present the compelling evidence that the design principle of the N-terminal specificity domain of mitochondrial presequences is fundamentally conserved among eukaryotes. Moreover, this domain might be derived from a TAT signal sequence, with subsequent modifications to enhance the efficiency of preprotein import into mitochondria during evolution. The results that hFH (human fumarate hydratase) presequence behaves as a fully functional presequence in plant cells and FA presequence (which is originated from Arabidopsis) can deliver GFP to the mitochondria in animal cells, strongly indicate the conservation of mitochondrial protein import mechanisms between plant and animal. Moreover, presequences from animal and fungi (which lack chloroplast) have the capability to deliver GFP to chloroplasts in plant cells when they lack multiple arginine residues (Lee et al., 2019). All these results strongly suggest the mechanistic conservation of mitochondrial import systems throughout the species in spite of some differences in the composition of Tom/Tim translocons among species (Mani et al., 2016). In the previous study, we showed the C-terminal domains of transit peptides and presequences are interchangeable to support the translocation across the membranes of both organelles (Lee et al., 2019). Interestingly, the C-terminal domain of presequences from human and fungi which do not contain chloroplasts supported the protein import into chloroplasts when the multiple arginine motif was removed from the N-terminal specificity domain of presequence (**Figure 1**), further suggesting the remarkable conservation of the design principle of presequences in eukaryotes.

Previously, we provided the possibility that during evolution diverse chloroplast transit peptides might have been generated through selective assembly of translocon component-interacting sequence motifs, which is consistent with the M&M (multi-selection and multi-order) model (Li and Teng, 2013; Lee et al., 2015). Then, where did N-terminal specificity domain of presequences come from? Here, we suggest that it might have been originated from the TAT signal sequence, considering the similarity in sequence motifs between the N-terminal specificity domain of presequences and TAT sequence. Both sequences contain multiple arginine residues and hydrophobic sequence motifs (Cline, 2015; Ulfig et al., 2017; Lee et al., 2019). In addition, incorporation of multiple arginine residues into transit peptides inhibit chloroplast protein import (Lee et al., 2019), which is also analogous to the temporary inhibition of secretion operated by the twin-arginine motif (Palmer and Berks, 2012). In the future, it will be necessary to elucidate how TAT signal sequences have been modified to achieve the efficient protein targeting to mitochondria.

## Data Availability Statement

All datasets generated for this study are included in the article/**Supplementary Material**.

## Author Contributions

DL and IH conceived this project. DL and SL carried out experiments in protoplasts. C-KM, CP, SP, and N-HC carried out experiments in mammalian cells. J-MK and C-SH carried out yeast transformation. DL and IH interpreted the data and wrote the manuscript.

## Conflict of Interest

The authors declare that the research was conducted in the absence of any commercial or financial relationships that could be construed as a potential conflict of interest.

## References

[B1] Abe Y. Shodai T. Muto T. Mihara K. Torii H. Nishikawa S. (2000). Structural basis of presequence recognition by the mitochondrial protein import receptor Tom20. Cell 100, 551–560. 10.1016/S0092-8674(00)80691-1 10721992

[B2] Alami M. Trescher D. Wu L. F. Muller M. (2002). Separate analysis of twin-arginine translocation (Tat)-specific membrane binding and translocation in Escherichia coli. J. Biol. Chem. 277, 20499–20503. 10.1074/jbc.M201711200 11923313

[B3] Cline K. (2015). Mechanistic Aspects of Folded Protein Transport by the Twin Arginine Translocase (Tat). J. Biol. Chem. 290, 16530–16538. 10.1074/jbc.R114.626820 25975269 PMC4505407

[B4] Dik E. Naamati A. Asraf H. Lehming N. Pines O. (2016). Human Fumarate Hydratase Is Dual Localized by an Alternative Transcription Initiation Mechanism. Traffic 17, 720–732. 10.1111/tra.12397 27037871

[B5] Dow J. M. Grahl S. Ward R. Evans R. Byron O. Norman D. G. (2014). Characterization of a periplasmic nitrate reductase in complex with its biosynthetic chaperone. FEBS J. 281, 246–260. 10.1111/febs.12592 24314029 PMC4159696

[B6] Dyall S. D. Brown M. T. Johnson P. J. (2004). Ancient invasions: from endosymbionts to organelles. Science 304, 253–257. 10.1126/science.1094884 15073369

[B7] Frobel J. Rose P. Muller M. (2011). Early Contacts between Substrate Proteins and TatA Translocase Component in Twin-arginine Translocation. J. Biol. Chem. 286, 43679–43689. 10.1074/jbc.M111.292565 22041896 PMC3243508

[B8] Garg S. G. Gould S. B. (2016). The Role of Charge in Protein Targeting Evolution. Trends Cell Biol. 26, 894–905. 10.1016/j.tcb.2016.07.001 27524662

[B9] Gietz R. D. Woods R. A. (2006). Yeast transformation by the LiAc/SS Carrier DNA/PEG method. Methods Mol. Biol. 313, 107–120. 10.1385/1-59259-958-3:107 16118429

[B10] Gray M. W. (2012). Mitochondrial evolution. Cold Spring Harb. Perspect. Biol. 4, a011403. 10.1101/cshperspect.a011403 22952398 PMC3428767

[B11] Gross J. Bhattacharya D. (2011). Endosymbiont or host: who drove mitochondrial and plastid evolution? Biol. Direct 6, 12. 10.1186/1745-6150-6-12 21333023 PMC3050876

[B12] Jin J. B. Kim Y. A. Kim S. J. Lee S. H. Kim D. H. Cheong G. W. (2001). A new dynamin-like protein, ADL6, is involved in trafficking from the trans-Golgi network to the central vacuole in Arabidopsis. Plant Cell 13, 1511–1525. 10.1105/TPC.000534 11449048 PMC139540

[B13] Lee D. W. Hwang I. (2011). Transient expression and analysis of chloroplast proteins in Arabidopsis protoplasts. Methods Mol. Biol. 774, 59–71. 10.1007/978-1-61779-234-2_4 21822832

[B14] Lee D. W. Kim J. K. Lee S. Choi S. Kim S. Hwang I. (2008). Arabidopsis nuclear-encoded plastid transit peptides contain multiple sequence subgroups with distinctive chloroplast-targeting sequence motifs. Plant Cell 20, 1603–1622. 10.1105/tpc.108.060541 18552198 PMC2483360

[B15] Lee S. Lee D. W. Yoo Y. J. Duncan O. Oh Y. J. Lee Y. J. (2012). Mitochondrial targeting of the Arabidopsis F1-ATPase gamma-subunit *via* multiple compensatory and synergistic presequence motifs. Plant Cell 24, 5037–5057. 10.1105/tpc.112.105361 23250447 PMC3556974

[B16] Lee D. W. Woo S. Geem K. R. Hwang I. (2015). Sequence motifs in transit peptides act as independent functional units and can be transferred to new sequence contexts. Plant Physiol. 169, 471–484. 10.1104/pp.15.00842 26149569 PMC4577419

[B17] Lee D. W. Lee S. Lee J. Woo S. Razzak M. A. Vitale A. (2019). Molecular mechanism of the specificity of protein import into chloroplasts and mitochondria in plant Cells. Mol. Plant 12, 951–966. 10.1016/j.molp.2019.03.003 30890495

[B18] Li H. M. Teng Y. S. (2013). Transit peptide design and plastid import regulation. Trends Plant Sci. 18, 360–366. 10.1016/j.tplants.2013.04.003 23688728

[B19] Li Y. Zheng X. X. Zhu M. H. Chen M. T. Zhang S. N. He F. Y. (2019). MoIVD-Mediated leucine catabolism is required for vegetative growth, conidiation and full virulence of the rice blast fungus Magnaporthe oryzae. Front. Microbiol. 10, 444. 10.3389/fmicb.2019.00444 30923517 PMC6426774

[B20] Mani J. Meisinger C. Schneider A. (2016). Peeping at TOMs-Diverse Entry Gates to Mitochondria Provide Insights into the Evolution of Eukaryotes. Mol. Biol. Evol. 33, 337–351. 10.1093/molbev/msv219 26474847

[B21] Palmer T. Berks B. C. (2012). The twin-arginine translocation (Tat) protein export pathway. Nat. Rev. Microbiol. 10, 483–496. 10.1038/nrmicro2814 22683878

[B22] Schleiff E. Becker T. (2011). Common ground for protein translocation: access control for mitochondria and chloroplasts. Nat. Rev. Mol. Cell Biol. 12, 48–59. 10.1038/nrm3027 21139638

[B23] Ulfig A. Frobel J. Lausberg F. Blummel A. S. Heide A. K. Muller M. (2017). The h-region of twin-arginine signal peptides supports productive binding of bacterial Tat precursor proteins to the TatBC receptor complex. J. Biol. Chem. 292, 10865–10882. 10.1074/jbc.M117.788950 28515319 PMC5491773

