# Supplementary material for: Cross-Species Functional Conservation and Possible Origin of the N-Terminal Specificity Domain of Mitochondrial Presequences
Source: Front Plant Sci. 2020 Feb 13;11:64. doi: 10.3389/fpls.2020.00064 (PMC7031408; doi:10.3389/fpls.2020.00064)
Supplement: Supplementary file 1 [file DataSheet_1.pdf]

A

```
NapA[N34]            : MKLSRRSFMKANAVAAAAAAAAGLSVPGVARAVVG
FA[N77]              : MAMAVFRREGRRLLPSIAARPIAAIRSPLSSDQEEGLLGVRSISTQVVRNRMKSVKNIQKITKAMKMVAASKLRAVQ
NapA[1-34]/FA[35-77] : MKLSRRSFMKANAVAAAAAAAAGLSVPGVARAVVGEGLLGVRSISTQVVRNRMKSVKNIQKITKAMKMVAASKLRAVQ
```

B

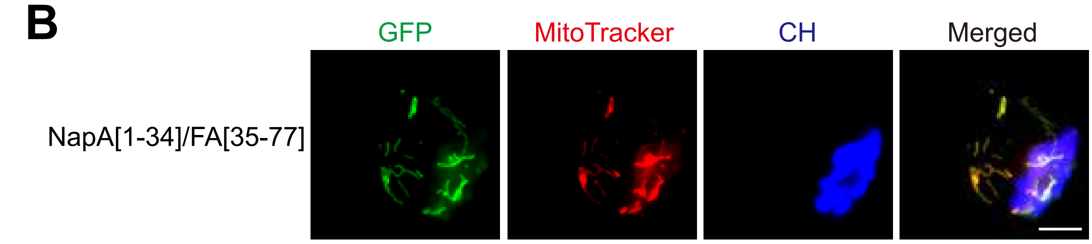

C

```
NapA[1-34]             : MKLSRRSFMKANAVAAAAAAAAGLSVPGVARAVVG
RbcS[N79]              : MASSMLSSATMVASPAQATMVAPFNGLKSSAAFPATRKANNDITSITSNGGRVNCMQVWPPIGKKKFETLSYLPDLTDS
PORA[N80]              : MALQAASLVSSAFSVRKDGKLNASASSSFKESSLFGVSLSEQSKADFVSSSLRCKREQSLRNNKAIIRAQAIATSTPSVT

NapA[1-34]/RbcS[35-79] : MKLSRRSFMKANAVAAAAAAAAGLSVPGVARAVVGATRKANNDITSITSNGGRVNCMQVWPPIGKKKFETLSYLPDLTDS
NapA[1-34]/PORA[35-80] : MKLSRRSFMKANAVAAAAAAAAGLSVPGVARAVVGFGVSLSEQSKADFVSSSLRCKREQSLRNNKAIIRAQAIATSTPSVT
```

D

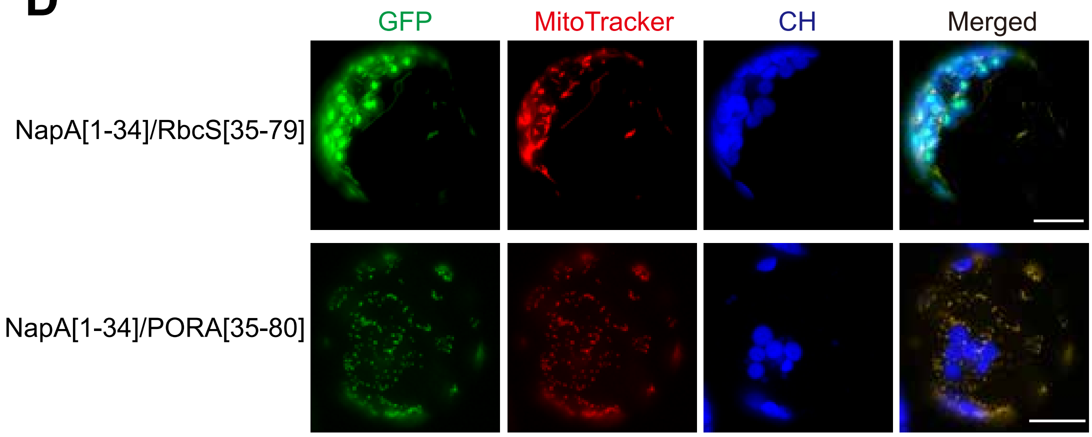

Supplementary Figure S1. NapA TAT sequence functionally replaces the mitochondrial specificity domain in presequences and confers mitochondrial specificity to transit peptides.

(A and C) Sequences of NapA[1–34], FA[N77], RbcS[N80], PORA[N80], and their hybrid mutants.
(B and D) Localization of reporter proteins. Green, red, and blue signals represent GFP, mitochondria stained with MitoTracker red, and chlorophyll autofluorescence, respectively. Scale bar = 20 µm.
